# Radiation leakage dose from Elekta electron collimation system

**DOI:** 10.1120/jacmp.v17i5.5982

**Published:** 2016-09-08

**Authors:** Garrett M. Pitcher, Kenneth R. Hogstrom, Robert L. Carver

**Affiliations:** ^1^ Department of Physics and Astronomy Louisiana State University Baton Rouge LA USA; ^2^ Mary Bird Perkins Cancer Center Baton Rouge LA USA

**Keywords:** therapeutic electron beams, electron collimation, electron Monte Carlo, radiation leakage dose

## Abstract

This study provided baseline data required for a greater project, whose objective was to design a new Elekta electron collimation system having significantly lighter electron applicators with equally low out‐of field leakage dose. Specifically, off‐axis dose profiles for the electron collimation system of our uniquely configured Elekta Infinity accelerator with the MLCi2 treatment head were measured and calculated for two primary purposes: 1) to evaluate and document the out‐of‐field leakage dose in the patient plane and 2) to validate the dose distributions calculated using a BEAMnrc Monte Carlo (MC) model for out‐of‐field dose profiles. Off‐axis dose profiles were measured in a water phantom at 100 cm SSD for 1 and 2 cm depths along the in‐plane, cross‐plane, and both diagonal axes using a cylindrical ionization chamber with the 10×10 and $20\times 20\;{\text{cm}}^{2}$ applicators and 7, 13, and 20 MeV beams. Dose distributions were calculated using a previously developed BEAMnrc MC model of the Elekta Infinity accelerator for the same beam energies and applicator sizes and compared with measurements. Measured results showed that the in‐field beam flatness met our acceptance criteria (±3% on major and ±4% on diagonal axes) and that out‐of‐field mean and maximum percent leakage doses in the patient plane met acceptance criteria as specified by the International Electrotechnical Commission (IEC). Cross‐plane out‐of‐field dose profiles showed greater leakage dose than in‐plane profiles, attributed to the curved edges of the upper X‐ray jaws and multileaf collimator. Mean leakage doses increased with beam energy, being 0.93% and 0.85% of maximum central axis dose for the 10×10 and $20\times 20\;{\text{cm}}^{2}$ applicators, respectively, at 20 MeV. MC calculations predicted the measured dose to within 0.1% in most profiles outside the radiation field; however, excluding modeling of nontrimmer applicator components led to calculations exceeding measured data by as much as 0.2% for some regions along the in‐plane axis. Using EGSnrc LATCH bit filtering to separately calculate out‐of‐field leakage dose components (photon dose, primary electron dose, and electron dose arising from interactions in various collimating components), MC calculations revealed that the primary electron dose in the out‐of‐field leakage region was small and decreased as beam energy increased. Also, both the photon dose component and electron dose component resulting from collimator scatter dominated the leakage dose, increasing with increasing beam energy. We concluded that our custom Elekta Infinity with the MLCi2 treatment head met IEC leakage dose criteria in the patient plane. Also, accuracy of our MC model should be sufficient for our use in the design of a new, improved electron collimation system.

PACS number(s): 87.56.nk, 87.10.Rt, 87.56.J

## I. INTRODUCTION

Four functions of the treatment head of radiotherapy accelerators used for electron beam therapy are: 1) to redirect the beam toward the patient, typically using an achromatic bending magnet; 2) to broaden and flatten the beam, typically using a dual scattering foil system; 3) to collimate the beam, typically using the X‐ray jaws and an electron applicator; and 4) to monitor beam output, flatness, and symmetry, typically using a dual, segmented, transmission ionization chamber.^(1)^ Current‐day dual scattering foil systems utilize uniform thickness primary foils to broaden the beam and approximately Gaussian‐shaped secondary foils to flatten the beam.^(2)^ Collimation systems consist of the X‐ray beam collimators, an electron applicator, and a custom applicator insert, which shapes the beam to the projection of the planning target volume (PTV) plus penumbral margin.

In our clinic, beam flatness is specified for off‐axis relative dose (normalized to 1.00 on central axis) 2 cm inside the field edges for all energy‐applicator combinations at a depth of 1.0 cm in water phantom for beams with most probable electron energies, $E_{p,0}\leq 9\;\text{MeV}$ and 2.0 cm for most probable electron energies, $E_{p,0}>9\;\text{MeV}$. Along major axes the relative dose should fall in the range of 1.000±0.030, and along diagonal axes 1.000±0.040; off‐axis beam symmetry should be within 2%.^3^, ^4^ Dose outside the irradiated volume of electron beams is specified to meet International Electrotechnical Commission (IEC) specifications for leakage dose.^(5)^ For electron therapy beams, IEC specifications state that: 1) the mean dose in air measured in the leakage region at patient plane (perpendicular to central beam axis at the normal treatment distance) should not exceed a value in the range of 1% to 1.8%, which varies with beam energy, of the maximum dose ($D_{\text{max}}$) on central axis at the normal treatment distance (100 cm SSD); 2) the maximum dose measured in the leakage region of the patient plane should not exceed 10% of $D_{\text{max}}$; and 3) the maximum dose measured at a position 2 cm outside the volume contained by the applicator should not exceed 10% of $D_{\text{max}}$.

Our clinical experience has shown that dose properties of electron beams on our customized Elekta (Elekta AB, Stockholm, Sweden) Infinity and standard Varian (Varian Medical Systems, Inc., Palo Alto, CA) Clinac radiotherapy accelerators, meet our beam uniformity and leakage specifications. However, we find Elekta electron applicators substantially heavier than comparable Varian applicators, as compared in Table 1, making their handling by our radiation therapy staff more difficult. Also, we find their latching mechanism for attaching the applicators to the treatment head awkward, and projection of the optical distance indicator (ODI) on the patient surface is obscured by the applicators, making setting the treatment SSD difficult. Hence, we believe there is clinical need to redesign the Elekta applicators and ODI to address these issues, and this study provided baseline data for a greater project funded by Elekta in which we designed a new Elekta electron collimation system having significantly lighter electron applicators with equally low out‐of‐field leakage dose.^(6)^

The Elekta dual scattering foil system has five primary foil positions on its first tray and three secondary foil positions on its second tray in the treatment head. The three secondary foils presently are required to cover the energy range of our clinical beams, 7–20 MeV; however, some accelerators with flattening filter‐free beams^(7)^ utilize one of the secondary foil positions.

**Table 1:** Comparison of Elekta and Varian electron applicator weights for five applicator sizes. The Elekta applicator sizes are specified at 95 cm SSD, and the Varian applicator sizes are specified projected to 100 cm SSD. The weight of the $15\times 15\;{\text{cm}}^{2}$ Varian applicator is listed in the $14\times 14\;{\text{cm}}^{2}$ column.

*Applicator Manufacturer*	*Applicator Weight (kg)*				
	$6\times 6\;{\text{cm}}^{2}$	$10\times 10\;{\text{cm}}^{2}$	$14\times 14\;{\text{cm}}^{2}$	$20\times 20\;{\text{cm}}^{2}$	$25\times 25\;{\text{cm}}^{2}$
Elekta	7.0	7.7	9.1	10.9	13.4
Varian	5.7	6.5	7.6^a^	8.6	9.5

^a^ Weight is for $15\times 15\;{\text{cm}}^{2}$ applicator.

In our clinic, this resulted in our relinquishing the use of our 16 and 20 MeV beams, which shared a common secondary foil.

Hence, radiotherapy practices with Elekta accelerators could benefit not only from redesign of the Elekta electron collimation system, but also its dual scattering foil system. Because the two systems have some interdependence, such redesign should be done together, as Varian^8^, ^9^ did to address its electron beams having both leakage issues^10^, ^11^, ^12^, ^13^, ^14^, ^15^ and beam uniformity issues along the diagonals of large fields.^(8)^ Scatter from collimating elements can impact central‐axis dose,^9^, ^16^ off‐axis dose flatness in the field,^17^, ^18^, ^19^ and leakage dose.^(20)^ Also, dual scattering foil design impacts the amount of X‐ray dose contamination and leakage dose, which is attenuated only a small amount by the thin electron applicator trimmers.

We have been investigating new electron scattering foil and collimation system designs for 6–20 MeV beams on the Elekta radiotherapy accelerator. Using a real‐time, dual scattering foil simulator,^(21)^ LeBlanc^(22)^ studied multiple dual scattering foil designs with results indicating that uniformity of 7–20 MeV beams using two secondary foils might be possible, demonstrating one secondary foil for 7–13 MeV and one for 16–20 MeV beams.

In parallel, we have been investigating a new electron beam collimation design, initially utilizing the current dual scattering foil system. Similarly, our investigation of scattering foil designs utilized the existing applicator design, although variation of the X‐ray jaw positions was allowed.^(22)^ Once both studies are completed, a combined solution might require small changes to be optimal.

Our first step in improving the design of the Elekta electron beam collimation system was to study the current system, which is the purpose of this study. More specifically, three goals of the present work were 1) to measure off‐axis dose, 2) to use the measured data to validate our Monte Carlo (MC) model of the Elekta electron beams for the calculation of off‐axis dose inside and outside the field, and 3) to use the MC model to calculate dose distributions from particles partitioned by type (electron or photon) and origin of scatter (X‐ray jaws, applicator trimmer bars, or air) for the 7, 13, and 20 MeV beams with the $20\times 20\;{\text{cm}}^{2}$ applicator. Results of the current study were paramount to a subsequent study, to be reported separately, that designed a new Elekta collimation system having significantly lighter applicators and equally acceptable in‐field dose flatness and out‐of‐field leakage dose.^(6)^

## II. MATERIALS AND METHODS

### A. Configuration of Accelerator

Our radiotherapy clinics utilize seven Elekta accelerators with matched electron beams meeting Mary Bird Perkins Cancer Center (MBPCC) customized clinical specifications, which differ slightly from those of standard Elekta beams. First, our beam energies of 7, 9, 10, 11, 13, 16, and 20 MeV ($E_{p,0}=7.1,8.7,9.9,11.3,13.1,16.2$, and 20.5 MeV) were selected for $R_{90}$ values with 0.5 cm (±0.1 cm) spacings of 2.0, 2.5, 3.0, 3.5, and 4.0 cm for 7–13 MeV beams and 1.0 cm spacings of 4.0, 5.0, and 6.0 cm for 13–20 MeV beams. These $R_{90}$ values differed by no more than 0.5 cm from those standardly offered by Elekta. Second, these small energy differences and our specifications for beam flatness of ±3% along major axes and ±4% along diagonals required slight modifications of standard dual scattering foils and X‐ray jaw settings.^22^, ^23^ Third, as typical in the United States, the standard treatment plane was 100 cm SSD, different from 95 cm SSD specified for Elekta applicators.

**Figure 1:**
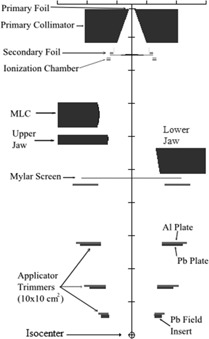
Cross‐sectional view of our uniquely configured Elekta Infinity electron treatment head (MLCi2) modeled in BEAMnrc with the $10\times 10\;{\text{cm}}^{2}$ applicator. The left side of the image demarcates the cross‐plane components (MLC and upper jaw); the right side of the image demarcates the in‐plane components (lower jaw). Beam central axis defines the z‐axis; z=0 is located at a point 100 cm upstream of isocenter. Tick marks have 10 cm spacing.

The current study was performed on one of six identically configured accelerators, the Elekta Infinity with the MLCi2 treatment head, which uses the in‐plane and cross‐plane X‐ray jaws, and attachable electron applicators to collimate its electron beams. Figure 1 shows a cross‐sectional view of the treatment head configuration created in BEAMnrc for electron beams with the $10\times 10\;{\text{cm}}^{2}$ applicator. The stationary tungsten primary collimator, positioned between the primary and secondary scattering foils, has little effect on electron beams. The primary scattering foils broaden the beam, and the secondary foils, flatten the beam.^21^, ^22^ The tungsten cross‐plane (upper) and in‐plane (lower) X‐ray collimators, or jaws, collimate the beam as it exits the treatment head. Both the multileaf collimator (MLC) and upper jaws are designed with curved inner edges, whereas the lower jaws are positioned to align with the divergence of the beam. These components are illustrated in Fig. 1, with the left side of the image depicting the cross‐plane and the right side of the image depicting the in‐plane collimating components. All other treatment head and collimation system components are 90° rotationally symmetrical. In electron mode the MLCs are parked in their farthest position off‐axis (±20 cm off central axis projected to isocenter in the cross‐plane direction with the treatment head at 0°) for all energies and applicators, and the underlying X‐ray jaws are used for initial collimation.

The MLCi2 treatment head in this study had five applicator sizes (6×6,10×10,14×14,20×20, and $25\times 25\;{\text{cm}}^{2}$ at 95 cm SSD). Each energy‐applicator combination had a unique in‐plane and cross‐plane X‐ray jaw setting adjusted to meet beam flatness and leakage requirements for our custom beam energies. Listed in Table 2 are the off‐axis X‐ray jaw positions projected to isocenter from a point on central axis located 100 cm upstream from isocenter for each nominal beam energy‐applicator combination investigated. These positions remained within Elekta's allowed ranges for which out‐of‐field doses were expected to meet IEC specifications.

The applicators utilize three trimmer levels. The upper two trimmers consist of a 0.6 cm thick layer of lead situated immediately beneath a 0.6 cm thick layer of aluminum. The lower trimmer consists of a 1 cm thick lead “open field” insert situated immediately beneath a 0.6 cm thick layer of aluminum. The lower face of the upper trimmer has a Z position of 73.3 cm for the four largest applicators and 77.2 cm for the $6\times 6\;{\text{cm}}^{2}$ applicator. The Z position is defined in this report as the distance along central axis from a point 100 cm upstream of isocenter. The lower faces of the middle trimmer and lower trimmer (field insert) have Z positions of 86.2 and 95.0 cm, respectively, for all applicator sizes. The inner edges of all trimmer plates are parallel to central axis, except for the inner edges of the “open field” inserts of the lower trimmer, which are divergent at an angle of 10° from central axis for all applicators.

**Table 2:** Off‐axis positions (cm) of X‐ray jaws (at isocenter) for the 7, 13, and 20 MeV nominal beam energies on the Elekta Infinity accelerator used in this study. The off‐axis positions of the jaw edges are projected to isocenter from a position 100 cm upstream of isocenter.

*Beam Parameters*	*Applicator*	*7 MeV*	*13 MeV*	*20 MeV*
In‐Plane Jaw Position (cm)	$10\times 10\;{\text{cm}}^{2}$	11.2	8.9	8.0
	$20\times 20\;{\text{cm}}^{2}$	14.3	12.7	11.7
Cross‐Plane Jaw Position (cm)	$10\times 10\;{\text{cm}}^{2}$	12.1	9.8	9.0
	$20\times 20\;{\text{cm}}^{2}$	15.3	13.5	12.8

Out‐of‐field leakage dose measurements for our uniquely configured electron beams should be comparable to those of comparable energy beams for other uniquely configured Elekta electron beams with the MLCi2 treatment head, but having slightly different dual scattering foils and custom X‐ray jaw settings within the limits allowed by Elekta. More importantly, validation of the MC dose calculations for our electron beams will allow utilization of the MC dose calculations as a tool for designing new Elekta electron beam collimation systems.^(6)^

### B. Measurements

Off‐axis dose profiles were measured to evaluate both beam flatness and patient plane leakage dose. Measurements were made at depths of 1 and 2 cm in a water phantom at 100 cm SSD for several beam energies and applicators on the Elekta Infinity accelerator at MBPCC. Resulting measurements were used to evaluate both the in‐field beam flatness and out‐of‐field leakage dose in the patient plane for each beam energy‐applicator combination. Beam flatness was evaluated using criteria specified by Hogstrom,^(4)^ and the leakage doses were evaluated according to specifications outlined by the IEC^(5)^ (described in detail in the Materials and Methods sections D.1 and D.2, respectively). Also, the measurements were used to assess the accuracy of a MC model created by Harris.^(23)^ Specifically, this was performed at shallow depths near the phantom surface where both the beam flatness and out‐of‐field leakage dose in the patient plane were evaluated.

### B.1 Scope of Measurements

Three beam energies, 7, 13, and 20 MeV, and two applicators, 10×10 and $20\times 20\;{\text{cm}}^{2}$, were selected for investigation. The 7, 13, and 20 MeV beams spanned the range of available energies, each using a separate one of the three secondary scattering foils. The IEC^(5)^ specifies that the mean and maximum percent leakage dose be determined for the lowest and highest available beam energies (7 and 20 MeV, respectively) on each accelerator.

Measurements were taken to comply with guidelines for determining field flatness according to Hogstrom,^(4)^ which provides detailed specifications based on American Association of Physicists in Medicine (AAPM) Report of Task Group 25 recommendations,^(3)^ and patient plane leakage dose levels according to the IEC.^(5)^ Scanned profiles were measured at 1 and 2 cm depth in a water phantom along the in‐plane, cross‐plane, and both diagonal axes for each beam energy‐applicator combination studied. Scans were performed from −24.5 to 24.5 cm off‐axis for all profiles measured with the $10\times 10\;{\text{cm}}^{2}$ applicator and the major axes profiles measured with the $20\times 20\;{\text{cm}}^{2}$ applicator, and from −29 to 29 cm off‐axis for the diagonal profiles measured with the $20\times 20\;{\text{cm}}^{2}$ applicator. These measured profiles provided all data necessary to evaluate both the in‐field beam flatness and out‐of‐field leakage dose in the patient plane.

### B.2 Methods of Measurements

Off‐axis ionization profiles were measured using an OmniPro two‐dimensional scanning tank with OmniPro (v. 6.2) scanning software package (Scanditronix Wellhofer AB RFA 20‐SERVO,

Uppsala, Sweden). Two N31011 cylindrical ionization chambers (PTW, Freiburg, Germany) were used for measurement, one for scanning and one for reference. These chambers had a 0.125 cm^3^ active volume with a length of 0.65 cm and an air cavity diameter of 0.55 cm. The scanning chamber was oriented with its stem perpendicular to both central axis of the beam and scanning direction. The beam scanning system was rotated 0°, 90°, and ±45° by rotating the treatment couch to measure the in‐plane, cross‐plane, and diagonal profiles, respectively. Between three and nine scans were taken of each off‐axis profile over the course of several different days. These data were validated by independent measurements for cross‐plane profiles at selected positions (0, 16, and 22 cm off‐axis at 1 cm depth) in solid water for energies of 7, 13, and 20 MeV with the $20\times 20\;{\text{cm}}^{2}$ applicator. The PTW Roos Model N34001 plane‐parallel chamber (PTW‐Freiburg) was used for these measurements with a Keithley model 614 electrometer (Keithley Instruments, Inc., Cleveland, OH). This chamber had an active volume of 0.35 cm^3^ (1.5 cm diameter collector and 0.2 cm electrode separation).

Each profile was normalized to the central‐axis ionization value at the depth of the scan, resulting in profiles of off‐axis relative ionization. These profiles were centered by shifting them small amounts such that the 50% relative ionization levels were equal distances from the central axis. Relative dose was assumed equal to relative ionization — that is to say, conversion factors from ionization to dose were assumed identical for central axis and all off‐axis positions. Using the multiple measurements for each condition, a mean and its standard error were calculated for each measurement point.

For the in‐field results, the mean measurement profiles were symmetrized by averaging each measured profile with the corresponding “mirrored” profile reflected about central axis. This reduced the effect of small beam asymmetries in evaluating the collimation system's beam flatness. The standard error of the mean for the symmetrized profile's data points was determined by summing in quadrature the fractional standard error of the mean at the two mirrored positions. For the out‐of‐field results, no symmetrization was performed.

For assessing in‐field beam flatness, the off‐axis relative dose profiles were utilized. For assessing out‐of‐field leakage, IEC specifications required dose measurements to be relative to central axis dose maximum, $D_{\text{max}}$. Hence, off‐axis relative dose values were multiplied by the central axis percent dose at 1 cm depth in water for the calculation of the mean and maximum percent leakage dose in the patient plane. Percent dose values at 1 cm were 95.2%, 94.5%, and 97.8% at 7, 13, and 20 MeV, respectively.

## C. MC calculations

This study used MC models created by Harris^(23)^ of the MBPCC configuration of Elekta Infinity electron therapy beams. The MLCi3 treatment head was modeled in BEAMnrc^(24)^ using manufacturer schematics of component geometries and materials, which were provided under a nondisclosure agreement. Simulations were performed using EGSnrc,^(25)^ and dose calculations were performed using DOSXYZnrc.^(26)^

## C.1 BEAMnrc model and source parameters

The treatment head components incorporated into the model included the vacuum exit window, primary scattering foil, primary collimator, secondary flattening foil, ionization monitor chambers, light field mirror, MLC, upper and lower X‐ray jaws, Mylar exit window, base plate for applicator attachment to the accelerator, applicator trimmer bars, and the applicator field insert.^22^, ^23^ The locations of these components are demarcated in Fig. 1. The structural components of the applicator, which attach the lower and middle trimmers, and the electrical components, which serve to code the inserts and trigger the beam interlock system if the applicator is interfered with during treatment, were excluded from the model due to their complex geometries and expectation of insignificant impact.

The incident electron source modeled by Harris^(23)^ was utilized; it produced central‐axis percent depth‐dose curves for the $25\times 25\;{\text{cm}}^{2}$ applicator that agreed within 2% or 0.1 cm of measured data. For the nominal 7, 13, and 20 MeV beams, the simulations were performed with $9\times 10^{8},4.5\times 10^{8}$, and $3\times 10^{8}$ initial electrons, respectively.

## C.2 Transport parameters and dose calculation

For MC calculations, the boundary crossing algorithm was set to PRESTA‐I, and the electron step algorithm was set to PRESTA‐II within BEAMnrc. Spin effects were turned on, and the maximum fractional energy loss was set to 0.25. The global cutoff energy was set to 0.521 MeV for electrons and 0.01 MeV for photons. Detailed explanations of the parameters used in this model and their functionality are provided by Harris^(23)^ and the BEAMnrc User's Manual.^(24)^

A phase space file, scored at a Z position of 99 cm, was created to store the particle type, position, directional cosines, energy, and normalization (relative to the original BEAMnrc source) information of every particle that reached the scoring plane. This phase space file was sampled by DOSXYZnrc to calculate dose in a water phantom. Particles within the phase space were recycled the necessary number of times to produce 10^9^ histories. DOSXYZnrc transported particles through 1 cm of air into a water phantom at 100 cm SSD. Dose was calculated in voxels (0.5 cm cube) within the water phantom centered at depths of 1 cm (extending from 0.75 cm to 1.25 cm depth) and 2 cm (extending from 1.75 to 2.25 cm depth). At each of these depths, a horizontal voxel matrix was created in which the centers of the voxels spanned from −24.5 to 24.5 cm off‐axis in both the in‐plane and cross‐plane dimensions. In the DOSXYZnrc model, all transport parameters were maintained from the BEAMnrc model, including the boundary crossing algorithm, electron step algorithm, spin effects setting, fractional energy loss, and global energy cutoffs. To reduce statistical uncertainty, the dose distributions were symmetrized in both the in‐plane and cross‐plane dimensions by reflecting them about central axis. Each profile was normalized to the mean dose calculated within a 3×3 set of voxels centered on central axis at the calculation depth of the profile.

Also, the LATCH bit filtering feature in EGSnrc was used to calculate dose from particles that interacted within various collimating components of interest. In this study, the bit filtering feature was used to tag particles with a particular component's LATCH bit if the particles interacted with that component, enabling dose from these particles to be calculated separately. The collimation components studied included the MLC, cross‐plane jaws, in‐plane jaws, and upper, middle, and lower trimmers. Additionally, total electron (photon) dose, that dose arising from electrons (photons) in the phase space 1 cm upstream of isocenter, was separately calculated. Dose components were calculated using DOSXYZnrc using the same phantom setup and phase space file inputs as the total dose calculation. These bit filtered calculations were performed for the $20\times 20\;{\text{cm}}^{2}$ applicator at energies of 7, 13, and 20 MeV. Mean percent leakage dose values were calculated for each component based on IEC specifications. This LATCH bit filtering methodology is similar to that used by Ebert and Hoban^(27)^ and Olsson,^(28)^ studies that focused primarily on the effects of scattered electrons on the in‐field and out‐of‐field dose distributions, respectively.

## D. Beam acceptance criteria

### D.1 In‐field beam flatness acceptance criteria

Beam flatness for both the measured and MC‐calculated dose within the field was evaluated according to the criteria described by Hogstrom,^(4)^ which we utilize in our clinic. The criteria state that off‐axis dose should not differ from the central‐axis dose by more than ±3% along the major axes (in‐plane and cross‐plane) and ±4% along the diagonal axes. These criteria apply to a region 2 cm inside the field edge for the major axes and 2√2 cm inside the corner edge of the field for the diagonal axes. These specifications, assessed at a depth of 1 cm in water for $E_{p,0}\leq 9\;\text{MeV}$ and 2 cm for $E_{p,0}>9\;\text{MeV}$, are a subset of those from AAPM Task Group 25,^(3)^ which states that flatness should be evaluated both near the surface and near the therapeutic depth.

### D.2 Out‐of‐field leakage dose acceptance criteria

**Figure 2:**
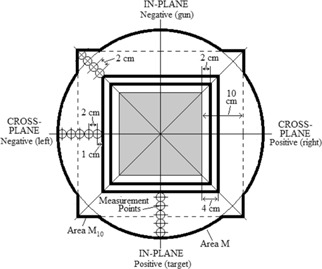
IEC‐specified geometry for measurements to obtain mean and maximum percent leakage dose. The diagram shows a beam's‐eye view of the $20\times 20\;{\text{cm}}^{2}$ applicator's radiation field projected to isocenter ($21.1\times 21.1\;{\text{cm}}^{2}$) with the surrounding leakage region defined by the IEC. The small circles with 2 cm spacing represent the points of dose evaluation within the leakage region at 1 cm depth in water below the isocentric plane. These points extend laterally from the inner boundary of the leakage region to the periphery of area M (black circle) or $M_{10}$ (thick black perimeter) along the in‐plane, cross‐plane and both diagonal axes. The inner boundary for determining mean leakage (outer thick black square) is 4 cm outside the radiation field. The inner boundary for determining maximum leakage (inner thick black square) is 2 cm outside the radiation field. The negative and positive off‐axis positions of the in‐plane (gun‐target) and cross‐plane (left‐right facing accelerator) axes are labelled. Adapted from IEC Report 60601‐2‐1.^(5)^

The out‐of‐field leakage dose was evaluated in the patient plane for both the measured and MC‐calculated dose distributions outside the field according to criteria described by the IEC.^(5)^ The IEC mandates that off‐axis profiles for determining the mean and maximum percent leakage dose be measured along the in‐plane and cross‐plane axes, as well as both diagonal axes at the treatment distance. From a practical perspective, it was assumed that off‐axis dose profiles at 1 cm depth in water (100 cm SSD) equaled off‐axis dose in air measured with a 1 cm buildup cap. We estimate these two values differ less than 0.02% of $D_{\text{max}}$. The in‐plane axis lies in the plane parallel and coincident to the plane of electron trajectory exiting the accelerating components and traveling through the bending magnets. The cross‐plane axis lies perpendicular to the in‐plane axis. The geometry illustrating the IEC measurement points is depicted in Fig. 2, which shows a beam's‐eye view of the isocentric plane (100 cm SSD) with the central shaded square representing the radiation field of the $20\times 20\;{\text{cm}}^{2}$ applicator ($21.1\times 21.1\;{\text{cm}}^{2}$ at isocenter). Measured profiles should extend laterally out to the edge of area M or $M_{10}$, whichever extent is applicable. Area M, defined as the geometric projection at isocenter of the primary collimator within the treatment head from a point 100 cm upstream of isocenter, has a radius of 24.8 cm at isocenter for the Elekta Infinity accelerator. Area $M_{10}$ is defined as the union of area M and a square with edges 10 cm outside the radiation field edges at isocenter. As the figure shows, for larger field sizes (e.g., $20\times 20\;{\text{cm}}^{2}$ Elekta applicator), measured profiles extend to the edge of area $M_{10}$. For small field sizes, such that a square with edges 10 cm outside the radiation field is completely contained within area M (e.g., $10\times 10\;{\text{cm}}^{2}$ Elekta applicator), the edge of area M defines the outer boundary of the measured profiles.

For calculation of the mean percent leakage dose, the inner boundary of the leakage region is defined by a square with edges located 4 cm outside the edge of the radiation field, indicated in Fig. 2 as the outer solid black square. For the calculation of the maximum leakage dose, the inner boundary of the leakage region is defined by a square with edges located 2 cm outside the radiation field, shown as the inner solid black square in the figure. For both mean and maximum leakage dose, measurements are evaluated at 2 cm spacing from the inner boundary to the border of area M or $M_{10}$, whichever is applicable, along the in‐plane, cross‐plane, and both diagonal axes in the patient plane (1 cm depth in water below the isocentric plane). The IEC mandates that the mean value of the leakage dose at these points not exceed a value in the range of 1% to 1.8% of $D_{\text{max}}$, which varies according to beam energy. The nominal beam energies used in this study, 7, 13, and 20 MeV, have specified mean leakage limits of 1.00%, 1.10%, and 1.34%, respectively (determined using $E_{p,0}$ values of 7.14, 13.12, and 20.47 MeV, respectively). The IEC also mandates that the maximum leakage dose of these measurement points not exceed 10% of $D_{\text{max}}$.

## III. RESULTS & DISCUSSION

### A. In‐field results

#### A.1 Evaluation of in‐field beam flatness

The in‐field major axes profiles of measured and MC‐calculated relative dose versus off‐axis position are plotted in 3, 4 for the 20×20 and $10\times 10\;{\text{cm}}^{2}$ applicators, respectively, for the 7, 13, and 20 MeV beams. The in‐field diagonal profiles are plotted in 5, 6 for the 20×20 and $10\times 10\;{\text{cm}}^{2}$ applicators, respectively, for the 7, 13, and 20 MeV beams. In 5, 6, the positive diagonal profiles refer to measurements scanned in the diagonal direction, which extends from the negative in‐plane and cross‐plane quadrant to the positive in‐plane and cross‐plane quadrant. The negative diagonal profiles refer to measurements scanned perpendicular to the positive diagonal profiles, extending from the positive in‐plane and negative cross‐plane quadrant to the negative in‐plane and positive cross‐plane quadrant. The 7 MeV profiles in these figures were measured at 1 cm depth, and the 13 and 20 MeV profiles were measured at 2 cm depth. Data points show the mean relative dose and the standard error of the mean relative dose calculated at each measurement point.

**Figure 3:**
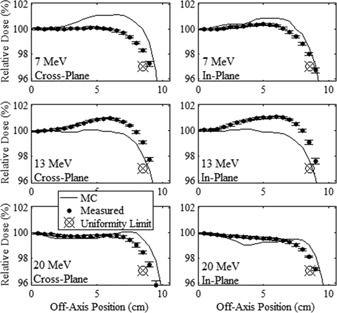
Comparison of measured and MC‐calculated in‐field major axes profiles of relative dose vs. off‐axis position for the $20\times 20\;{\text{cm}}^{2}$ applicator. Cross‐plane and in‐plane profiles are compared at beam energies of 7 (upper row), 13 (middle row), and 20 MeV (lower row). The 7 MeV beam profiles were measured at 1 cm depth in water, and the 13 and 20 MeV profiles were measured at 2 cm depth. Solid circles represent the mean measured profiles with error bars indicating the standard error of the mean. MC‐calculated profiles, represented by the solid curves, have statistical uncertainty of approximately 0.1% of central‐axis dose. The uniformity limit marker (⊗) represents the minimum relative dose required to pass the minimum dose flatness criteria at the edge of the uniformity region.

**Figure 4:**
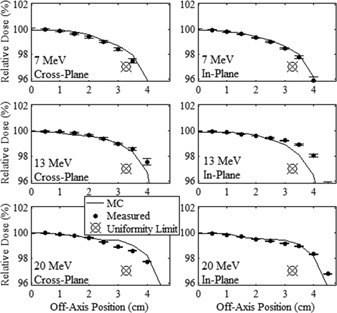
Comparison of measured and MC‐calculated in‐field major axes profiles of relative dose vs. off‐axis position for the $10\times 10\;{\text{cm}}^{2}$ applicator, with the same comparisons and measurement conditions as for Fig. 3.

**Figure 5:**
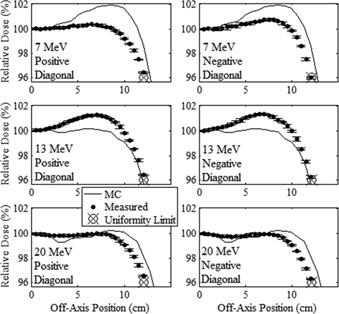
Comparison of measured and MC‐calculated in‐field diagonal axes profiles of relative dose vs. off‐axis position for the $20\times 20\;{\text{cm}}^{2}$ applicator. Positive and negative diagonal profiles are compared at beam energies of 7 (upper row), 13 (middle row), and 20 MeV (lower row) with the same comparisons and measurement conditions as Fig. 3.

**Figure 6:**
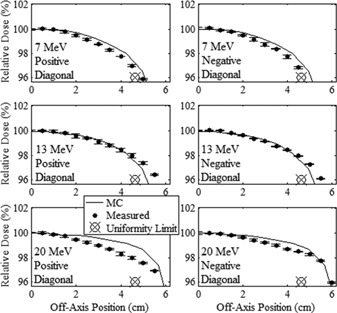
Comparison of measured and MC‐calculated in‐field diagonal axes profiles of relative dose vs. off‐axis position for the $10\times 10\;{\text{cm}}^{2}$ applicator. Positive and negative diagonal profiles are compared at beam energies of 7 (upper row), 13 (middle row), and 20 MeV (lower row) with the same comparisons and measurement conditions as Fig. 3.

3, 6 show the flatness of the beam within the field, with the uniformity limit markers representing the minimum relative dose at the edge of the uniformity region (the lateral region over which the beam uniformity is evaluated for each off‐axis profile) required to pass our beam flatness criteria. These markers are positioned at relative doses of 97% and 96% for the major axes and diagonal axes, respectively, at the edge of the uniformity region. Profiles which pass above and outside this marker are deemed acceptably flat with respect to the minimum dose specification in the uniformity region. The figures show that this is true for all measured profiles, indicating that all investigated measured beams passed beam flatness minimum dose criteria at this off‐axis position. Also, the plots show the maximum off‐axis doses are less than 101.5% for all profiles, well within the flatness maximum dose of 103%.

The minimum and maximum differences of measured off‐axis relative dose minus central‐axis dose, expressed as (minimum difference, maximum difference), within the uniformity region of each profile are listed in Table 3. For all major axes profiles the measured relative dose falls within ±3% of central‐axis dose, and for all diagonal profiles the measured relative dose falls within ±4% of central axis dose, passing flatness criteria. The positive and negative measured diagonal profiles for the 7 MeV beam with the $20\times 20\;{\text{cm}}^{2}$ applicator narrowly pass flatness criteria, with minimum doses of 96.1% and 96.0% (3.9% and 4.0%, respectively, less than central axis dose) within the uniformity region. The significance of these data is that low out‐of‐field leakage dose has not come at the expense of beam flatness.

**Table 3:** Minimum and maximum differences of measured off‐axis relative dose from central‐axis relative dose, expressed as (minimum difference, maximum difference), within the uniformity region. The 7 MeV differences were determined at 1 cm depth in water, and the 13 and 20 MeV differences were determined at 2 cm depth.

*Profile Dimension*	*7 MeV*	*13 MeV*	*20 MeV*
$10\times 10\;{\text{cm}}^{2}$ Applicator			
In‐Plane	(−1.6%,0.0%)	(−0.8%,0.0%)	(−0.9%,0.0%)
Cross‐Plane	(−1.6%,0.0%)	(−1.0%,0.0%)	(−1.1%,0.0%)
Diagonal (Pos)^a^	(−3.4%,0.0%)	(−2.3%,0.0%)	(−2.2%,0.0%)
Diagonal (Neg)^b^	(−3.2%,0.1%)	(−2.0%,0.1%)	(−1.5%,0.0%)
$20\times 20\;{\text{cm}}^{2}$ Applicator			
In‐Plane	(−2.3%,0.4%)	(−0.9%,1.1%)	(−1.9%,0.0%)
Cross‐Plane	(−1.8%,0.2%)	(−0.9%,1.0%)	(−1.6%,0.0%)
Diagonal (Pos)^a^	(−3.9%,0.4%)	(−3.6%,1.3%)	(−3.4%,0.0%)
Diagonal (Neg)^b^	(−4.0%,0.8%)	(−3.7%,1.4%)	(−3.7%,0.0%)

^a^ Positive diagonal profile extends from negative in‐plane and cross‐plane quadrant to positive in‐plane and cross‐plane quadrant.

^b^ Negative diagonal profile runs perpendicular to positive diagonal profile.

#### A.2 Comparison of MC‐calculated and measured data

3, 6 show that the MC calculations mostly slightly overpredicted the in‐field measured dose for both the 7 and 20 MeV beams, with the 7 MeV beam having the greater discrepancy, and that the MC calculations slightly underpredicted the measured dose for the 13 MeV beam. Also, the differences were less for the $10\times 10\;{\text{cm}}^{2}$ applicator with data points not being as far off‐axis as for the $20\times 20\;{\text{cm}}^{2}$ applicator. The maximum differences in the MC‐calculated minus measured dose within the uniformity region of each profile are listed in Table 4 for each beam energy‐applicator combination studied. These results show that the 7 and 20 MeV MC‐calculated doses overpredicted the measured doses by as much as 2.7% and 2.3%, respectively, and the 13 MeV MC‐calculated dose underpredicted the measured dose by as much as 1.7%, all results of the $20\times 20\;{\text{cm}}^{2}$ applicator. These results for in‐field agreement of MC‐calculated and measured off‐axis dose near the surface are consistent with the findings of Harris.^(23)^

**Table 4:** Maximum differences in the MC‐calculated and measured relative dose (MC minus measured) within the uniformity region for each profile. The differences are listed as percentage of central‐axis dose at the depth of measurement.

*Profile Dimension*	$10\times 10\;{\text{cm}}^{2}$ Applicator			$20\times 20\;{\text{cm}}^{2}$ Applicator		
	*7 MeV*	*13 MeV*	*20 MeV*	*7 MeV*	*13 MeV*	*20 MeV*
In‐Plane	0.4%	−0.6%	0.3%	0.9%	−1.7%	1.0%
Cross‐Plane	0.4%	−0.2%	0.5%	1.9%	−1.3%	1.4%
Diagonal (Pos)^a^	1.0%	−0.3%	1.2%	2.6%	−1.5%	2.3%
Diagonal (Neg)^b^	0.8%	−0.3%	1.1%	2.7%	−1.7%	2.2%

^a^ Positive diagonal profile extends from negative in‐plane and cross‐plane quadrant to positive in‐plane and cross‐plane quadrant.

^b^ Negative diagonal profile runs perpendicular to positive diagonal profile.

### B. Out‐of‐field results

#### B.1 Evaluation of patient plane leakage dose

**Figure 7:**
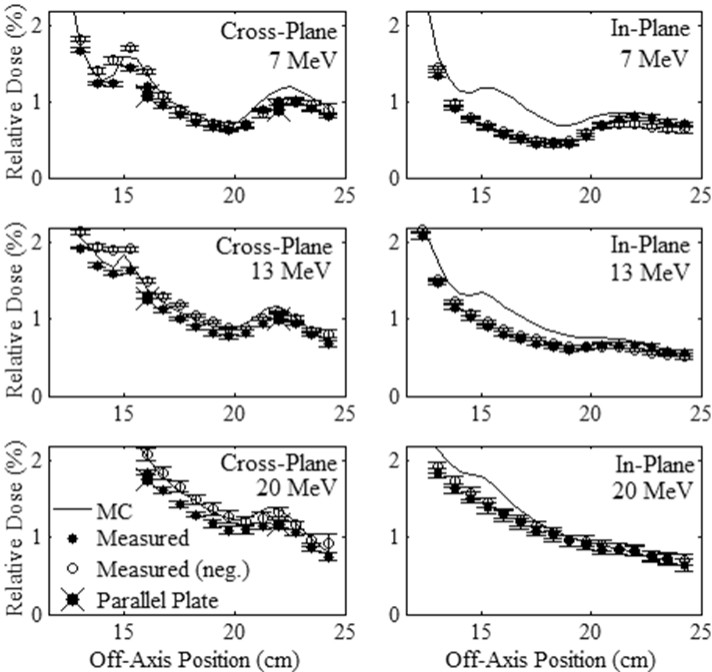
Comparison of measured and MC‐calculated out‐of‐field major axes profiles of relative dose vs. off‐axis position for the $20\times 20\;{\text{cm}}^{2}$ applicator. Cross‐plane and in‐plane profiles are compared at beam energies of 7 (upper row), 13 (middle row), and 20 MeV (lower row). All profiles were normalized to central axis dose at the depth of measurement, 1 cm in water. The solid circles represent the mean measured profiles in the positive direction, and the open circles represent the same profiles in the negative direction, mirrored about central axis. The error bars indicate the standard error of the mean for each measurement point. The MC‐calculated profiles, represented by the solid curves, have statistical uncertainty of approximately 0.01% of central‐axis dose. Select parallel plate ionization chamber measurement points in the cross‐plane profiles validated the cylindrical ionization chamber measurements.

The measured and MC‐calculated profiles of relative dose versus off‐axis position along the major axes in the out‐of‐field leakage region are plotted in 7, 8 for the 20×20 and $10\times 10\;{\text{cm}}^{2}$ applicators, respectively. The measured and MC‐calculated relative dose profiles versus off‐axis position along the positive diagonal axes are plotted in Fig. 9 for the two applicators. The measured and MC‐calculated negative diagonal profiles were symmetrically equivalent and matched the positive diagonal profiles and, for this reason, were omitted from plotting. All profiles were normalized to central axis dose at the depth of measurement, 1 cm in water. Figure 7 also plots the parallel plate ionization chamber measurement points in the cross‐plane profile for the $20\times 20\;{\text{cm}}^{2}$ applicator, which validated the cylindrical ionization chamber measurements, agreeing within 0.11%.

Measured in‐plane leakage doses are lower, by as much as a factor of two, than cross‐plane leakage doses. In general, these differences are greater for greater off‐axis distances (20–25 cm), the smaller applicator ($10\times 10\;{\text{cm}}^{2}$), and lower energies. These differences are attributed primarily to there being more electrons scattered off the curved MLC and upper X‐ray jaw edges, which collimate the beam in the cross‐plane direction. Electron scatter is less from the lower X‐ray jaws, which collimate in the in‐plane direction and have straight collimation edges diverging from a point 100 cm above isocenter. This premise is supported by MC analysis of scattered electrons in the Results section B.2.

**Figure 8:**
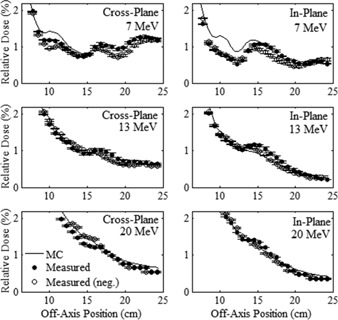
Comparison of measured and MC‐calculated out‐of‐field major axes profiles of relative dose versus off‐axis position for the $10\times 10\;{\text{cm}}^{2}$ applicator, with the same comparisons and measurement conditions as for Fig. 7.

**Figure 9:**
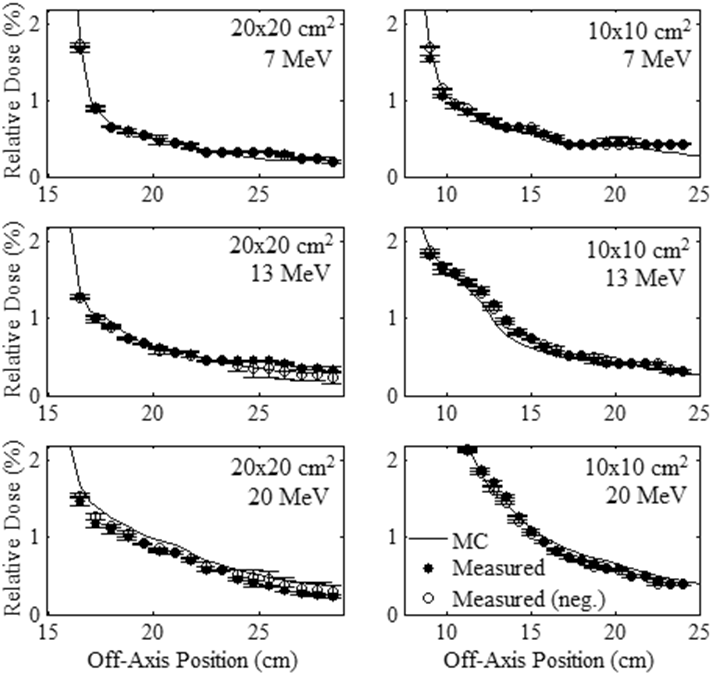
Comparison of measured and MC‐calculated out‐of‐field positive diagonal axes profiles of relative dose vs. off‐axis position. The 20×20 and $10\times 10\;{\text{cm}}^{2}$ applicator profiles are compared at beam energies of 7 (upper row), 13 (middle row), and 20 MeV (lower row) with the same comparisons and measurement conditions as Fig. 7.

Mean percent leakage doses at the patient plane, calculated for each beam according to the procedure specified by the IEC, are listed in Table 5 for both the measured and MC‐calculated data. The percentages in this table are normalized to $D_{\text{max}}$, as specified by the IEC. Results revealed that the accelerator met IEC specified standards for patient plane mean leakage for all beam energy‐applicator combinations studied, with measured mean percent leakage doses being well less than the IEC specified maxima of 1.00%, 1.10%, and 1.34% for the 7, 13, and 20 MeV beams, respectively. In general the measured mean leakage dose was greatest for the 20 MeV beam, being 0.93% for the $10\times 10\;{\text{cm}}^{2}$ applicator. Also, our results were consistent with MBPCC clinical commissioning measurements, which showed that MBPCC Elekta electron beams produced clinically acceptable in‐field beam flatness for the $25\times 25\;{\text{cm}}^{2}$ applicator and out‐of‐field leakage dose for all applicators at all seven beam energies.

The maximum percent leakage doses are listed in Table 6 as a percentage of $D_{\text{max}}$, per IEC specifications for both measured and MC‐calculated data. Results revealed that the maximum leakage for all beams was well below the IEC specified threshold of 10%. The maximum dose for all beams was 4.38% for the MC‐calculated data and 4.09% for the measured data, found in the cross‐plane profile of the 20 MeV beam with the $10\times 10\;{\text{cm}}^{2}$ applicator.

Comparing our 7 and 13 MeV Elekta leakage dose profiles for the $10\times 10\;{\text{cm}}^{2}$ applicator to 6 and 12 MeV leakage dose profiles reported by Shimozato et al.^(20)^ for the Varian Clinac 2100CD showed Elekta leakage doses to be greater. Because the profiles in the Shimozato study were measured at 0.5 cm depth, comparing them to our profiles at 1.0 cm depth required that they be scaled by the ratios of dose at a depth of 1.0 cm to that at 0.5 cm (± 0.7 and 0.8 at 15 cm off‐axis for Varian 6 and 12 MeV beams, respectively, using measured data reported by Cardenas et al.^(29)^). At 20 cm off‐axis, the Elekta in‐plane and cross‐plane leakage doses were approximately two and four times, respectively, those of the Shimozato study comparing 7 and 6 MeV beams, and approximately equal and two times, respectively, those of the Shimozato study comparing 13 and 12 MeV beams.

Comparing our 7, 13, and 20 MeV Elekta leakage dose profiles at 15–25 cm off‐axis for the $10\times 10\;{\text{cm}}^{2}$ and $20\times 20\;{\text{cm}}^{2}$ applicators to 6, 12, and 18 MeV leakage dose profiles reported by Yeboah et al.^(30)^ for the Siemens EA200 electron applicators showed Elekta leakage doses to be less for most cases. At 7 MeV, Elekta leakage doses were comparable to Yeboah and colleagues' leakage doses, except for being approximately 2.5 times greater 25 cm off‐axis for the $10\times 10\;{\text{cm}}^{2}$ applicator and 15 cm off‐axis for the $20\times 20\;{\text{cm}}^{2}$ applicator. At 20 MeV, Elekta leakage doses were always less, being approximately 0.25–0.5 those of Yeboah et al. for the $10\times 10\;{\text{cm}}^{2}$ applicator and 0.2–0.6 those for the $20\times 20\;{\text{cm}}^{2}$ applicator; at 13 MeV, Elekta leakage doses were 0.4–1.0 those of Yeboah et al. The differences in magnitude and shape of the leakage dose profiles for different manufacturers illustrate the impact that differences in collimation systems (X‐ray jaws and applicators) can have.

**Table 5:** Mean percent leakage doses calculated as a percentage of $D_{\text{max}}$ per IEC specifications for measured and MC‐calculated off‐axis dose profiles. The maximum mean percent leakage dose values allowed by the IEC are listed for each beam energy.

*Beam Energy*	*IEC Specified Maximum*	*Measured*		*MC*		*Difference (MC ‐ Measured)*	
		$10\times 10\;{\text{cm}}^{2}$	$20\times 20\;{\text{cm}}^{2}$	$10\times 10\;{\text{cm}}^{2}$	$20\times 20\;{\text{cm}}^{2}$	$10\times 10\;{\text{cm}}^{2}$	$20\times 20\;{\text{cm}}^{2}$
7 MeV	1.00%	0.66%	0.56%	0.70%	0.67%	0.04%	0.11%
13 MeV	1.10%	0.67%	0.65%	0.65%	0.69%	−0.02%	0.04%
20 MeV	1.34%	0.93%	0.85%	0.99%	0.93%	0.06%	0.08%

**Table 6:** Maximum percent leakage doses calculated as a percentage of $D_{\text{max}}$ per IEC specifications for measured and MC‐calculated off‐axis dose profiles.

*Beam Energy*	*Measured*		*MC*		*Difference (MC ‐ Measured)*	
	$10\times 10\;{\text{cm}}^{2}$	$20\times 20\;{\text{cm}}^{2}$	$10\times 10\;{\text{cm}}^{2}$	$20\times 20\;{\text{cm}}^{2}$	$10\times 10\;{\text{cm}}^{2}$	$20\times 20\;{\text{cm}}^{2}$
7 MeV	2.14%	1.74%	2.11%	1.65%	−0.03%	−0.09%
13 MeV	2.78%	2.03%	2.75%	1.95%	−0.03%	−0.08%
20 MeV	4.09%	2.73%	4.38%	2.83%	0.29%	0.10%

#### B.2 Comparison of MC‐calculated and measured data

7, 8, 9 show that the MC‐calculated dose profiles agreed well with the measured out‐of‐field dose profiles, with the only notable differences being for the in‐plane profiles near the edge of the field where the MC calculations overpredicted the measured dose. These differences were greatest for the $20\times 20\;{\text{cm}}^{2}$ applicator and 7 MeV beam. As previously described, this discrepancy is attributed to the structural and electrical components, which are mostly present between the middle and lower trimmers along the in‐plane sides of the applicator, being omitted from the MC model.

Table 7 shows the minimum and maximum differences of the MC‐calculated minus measured relative dose, expressed as (minimum difference, maximum difference), for each profile in the leakage region. MC‐calculations overpredicted the measured data in the in‐plane dimension by a maximum difference of 0.56%, 0.41%, and 0.33% for the $20\times 20\;{\text{cm}}^{2}$ applicator at 7, 13, and 20 MeV, respectively. The maximum difference for the $10\times 10\;{\text{cm}}^{2}$ applicator was 0.50% at 7 MeV. For all other profiles the MC‐calculations agree with the measured data within 0.26%. This level of agreement is similar to a previous study by Shimozato et al.^(20)^ in which MC data calculated using a BEAMnrc model was compared with cylindrical ionization chamber measurements in the same region for a Varian Clinac 2100CD accelerator, which had a maximum difference of 0.23% for a $10\times 10\;{\text{cm}}^{2}$ applicator.

The MC calculations slightly overpredicted the measured mean percent leakage dose by an average of 0.05%, but never more than 0.11%. Again, we attribute this small difference to possibly excluding the structural and electrical applicator components in the applicator model used for MC calculations.

**Table 7:** Minimum and maximum differences of the MC‐calculated minus measured relative dose, expressed as (minimum difference, maximum difference), for each off‐axis profile in the leakage region. Values are calculated as a percent of central‐axis dose at 1 cm depth.

*Profile Dimensions*	*7 MeV*	*13 MeV*	*20 MeV*
$10\times 10\;{\text{cm}}^{2}$ Applicator			
In‐Plane	(−0.07%,0.50%)	(−0.24%,0.13%)	(−0.15%,0.20%)
Cross‐Plane	(−0.19%,0.24%)	(−0.18%,0.17%)	(−0.25%,0.26%)
Diagonal (Pos)^a^	(−0.18%,0.05%)	(−0.13%,0.02%)	(−0.10%,0.13%)
Diagonal (Neg)^b^	(−0.13%,0.00%)	(−0.14%,0.01%)	(−0.02%,0.13%)
$20\times 20\;{\text{cm}}^{2}$ Applicator			
In‐Plane	(−0.05%,0.56%)	(−0.01%,0.41%)	(−0.04%,0.33%)
Cross‐Plane	(−0.07%,0.20%)	(−0.14%,0.16%)	(−0.10%,0.22%)
Diagonal (Pos)^a^	(−0.07%,0.01%)	(0.08%,−0.01%)	(−0.07%,0.12%)
Diagonal (Neg)^b^	(−0.07%,0.02%)	(−0.07%,0.03%)	(0.01%, 0.12%)

^a^ Positive diagonal profile extends from negative in‐plane and cross‐plane quadrant to positive in‐plane and cross‐plane quadrant.

^b^ Negative diagonal profile runs perpendicular to positive diagonal profile.

To gain further understanding of the sources of out‐of‐field leakage dose, MC calculations were performed using the LATCH bit filtering feature of EGSnrc to separately calculate the distributions from different leakage dose components. 10, 11 display the results of these calculations, plotting the cross‐plane and in‐plane leakage dose profiles, respectively, for each dose component at 1 cm depth in water for the $20\times 20\;{\text{cm}}^{2}$ applicator for the 7, 13, and 20 MeV beams. Plots in the left column show the total leakage dose, photon leakage dose, primary electron leakage dose, and scattered electron leakage dose. Primary electron leakage dose arises from electrons that had no interactions with the collimation components. Scattered electron dose, calculated as the total electron dose minus the primary electron dose, is partitioned into dose from electrons that arise from interactions (photon and electron) in the various collimation system components, including the MLC, upper jaw, lower jaw, and the upper, middle, and lower trimmers. The leakage dose profiles from each component are plotted in the right column. Comparing, in‐plane leakage dose profiles are less than cross‐plane leakage dose profiles, primarily due to there being less scattered electron leakage dose, as photon leakage dose profiles are comparable, and primary electron leakage dose profiles are greater. Furthermore, analysis of the origin of scattered electron dose shows the difference is primarily due to reduced photon jaw scatter, particularly that of the MLC and upper photon jaws, which have curved edges.

Using these MC‐calculated out‐of‐field dose distributions, the mean percent leakage doses from each component were calculated as a percentage of $D_{\text{max}}$ per IEC specifications and are listed in Table 8. The results indicate the total percent mean leakage dose increases slightly from 7 to 13 MeV, but significantly from 13 to 20 MeV. This is primarily due to the photon dose increase, most likely due to increased bremsstrahlung from the scattering foils with energy. The primary electron leakage dose, which represents electrons scattered by air and not by the collimating components, is small, indicating relatively few electrons take tortuous paths between trimmers to contribute out‐of‐field dose in the patient plane. Hence, the total electron percent mean leakage dose comes primarily from electron and photon interactions in the collimating system components scattering electrons to the patient plane. Further investigation is required to determine what portion of this dose is due to electrons scattered from the edges of the collimation system and from electrons produced within the collimation system by photons undergoing photoelectric or Compton interactions. Table 8 and 10, 11 show that the majority of the electron leakage dose came from interactions with the applicator trimmers, with the middle and lower trimmer scatter dose components increasing with increasing energy, and the upper trimmer dose component decreasing with increasing energy.

**Figure 10:**
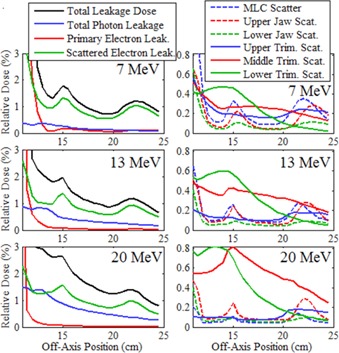
Cross‐plane relative leakage dose component profiles for the $20\times 20\;{\text{cm}}^{2}$ applicator plotted vs. off‐axis position. Beam profiles at 7 MeV (top row), 13 MeV (middle row), and 20 MeV (bottom row) are calculated at 1 cm depth in water. The left column of plots subdivides the total leakage dose (black) into its primary electron (red), scattered electron (green), and total photon (blue) components. The right column further subdivides the scattered electron leakage dose into electrons scattered from various collimation components.

**Figure 11:**
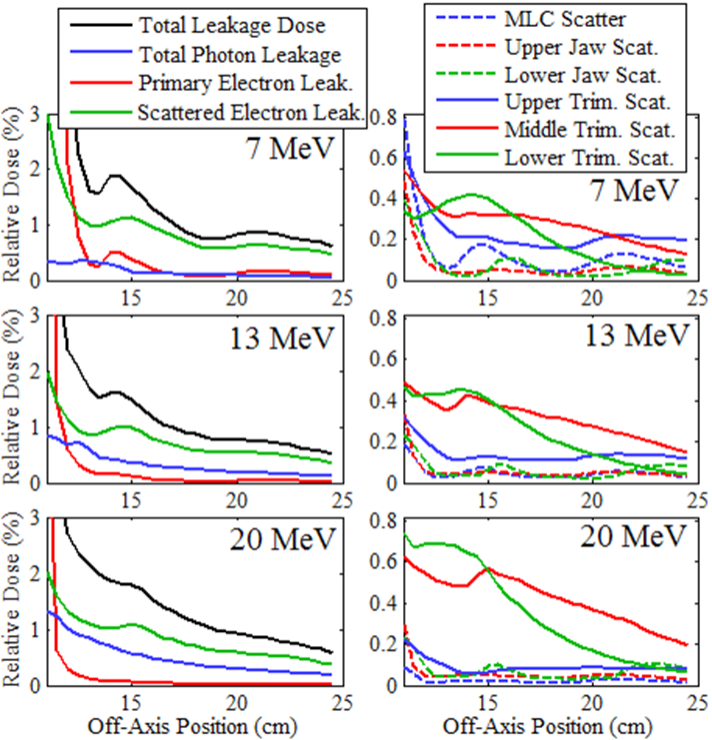
In‐plane relative leakage dose component profiles for the $20\times 20\;{\text{cm}}^{2}$ applicator plotted vs. off‐axis position with the same comparisons and calculation conditions as Fig. 10.

**Table 8:** Contributions to percent mean leakage dose for the $20\times 20\;{\text{cm}}^{2}$ applicator at 7, 13, and 20 MeV. Mean percent leakage dose contributions were independently calculated for each particle type (electron and photon) incident on a water phantom at 100 cm SSD, for those electrons that interact with no collimation components (primary electrons), and for those electrons that arise from interactions in each of the six collimating components. All percent mean leakage dose values were calculated per IEC specifications ($100\%=D_{\text{max}}$).

*Leakage Dose Contributions*	*7 MeV*	*13 MeV*	*20 MeV*
Total Leakage Dose	0.67%	0.69%	0.93%
Total Electron	0.58%	0.49%	0.56%
Total Photon	0.09%	0.20%	0.34%
Primary Electrons	0.08%	0.02%	0.02%
MLC Scatter	0.10%	0.07%	0.03%
Upper Jaw Scatter	0.06%	0.06%	0.06%
Lower Jaw Scatter	0.04%	0.04%	0.05%
Upper Trimmer Scat.	0.13%	0.09%	0.07%
Middle Trimmer Scat.	0.16%	0.21%	0.35%
Lower Trimmer Scat.	0.11%	0.13%	0.17%

## IV. CONCLUSIONS

MBPCC Elekta electron beams meet the in‐field flatness criteria of Hogstrom,^(4)^ which is a subset of that of AAPM Report of Task Group 25,^(3)^ and out‐of‐field leakage dose criteria in the patient plane, based on the IEC^(5)^ specifications, for the 10×10 and $20\times 20\;{\text{cm}}^{2}$ applicators at 7, 13, and 20 MeV. Data from the current study provide a quality benchmark for future improved designs of the electron collimation system with reduced applicator weights.^(6)^

Other Elekta Infinity accelerators with the MLCi2 treatment head, but with Elekta's standard beam energies, photon jaw positions, and scattering foils, should have similar, but slightly different, flatness and leakage doses. Also, our out‐of‐field leakage results do not apply to the newer Agility treatment head, which utilizes curved MLC ends and curved X‐ray jaw ends to collimate the beam in the cross‐plane and in‐plane directions, respectively, both different from the MLCi2 X‐ray jaw configuration.

MC‐calculated doses based on this study's model showed sufficient accuracy to be useful for the design of new improved electron collimation and dual scattering foil systems. Although in‐field dose calculations differed from measurements by as much as 2.7% in some regions, similar to the results of Harris,^(23)^ these small differences can be accounted for in an optimization process using a technique in which the objective function is modified accordingly, as demonstrated by LeBlanc^(22)^ in designing potentially new Elekta scattering foils. Additionally, MC‐calculated dose is sufficiently accurate for assessment of out‐of‐field leakage dose. IEC‐specified mean leakage doses agreed within 0.1%, and maximum leakage doses agreed within 0.3% of measured data.

Analysis of MC‐calculated out‐of‐field leakage dose showed that the electron dose component was greater than photon dose, that the photon dose component became increasingly important as energy increased, and that the electron dose came almost entirely from electrons scattered from all collimation system components, particularly the trimmers of the electron applicators. Also, differences between in‐plane and cross‐plane leakage dose profiles demonstrate the significance of photon jaw positioning and shape of its collimating edges. Hence, with respect to out‐of‐field leakage dose, the impact of scattered electron dose is important to the design of improved collimation systems.

## ACKNOWLEDGMENTS

This research was funded in part through a research agreement with Elekta Limited. Portions of this research were conducted with high performance computational resources provided by Louisiana State University (http://www.hpc.lsu.edu).

## COPYRIGHT

This work is licensed under a Creative Commons Attribution 3.0 Unported License.
